# Efficacy and safety of acupuncture for elderly patients with coronavirus disease 2019 (COVID-19): A protocol for systematic review and meta-analysis

**DOI:** 10.1097/MD.0000000000024515

**Published:** 2021-02-05

**Authors:** Qingchang Xia, Huawei Gao, Jin Xian, Xiao Yan, Yue Zhou, Yunping Lu, Yuxia Ma

**Affiliations:** College of Acupuncture and Massage, Shandong University of Traditional Chinese Medicine, Jinan, Shandong, China.

**Keywords:** acupuncture, coronavirus disease 2019, elderly patients, protocol, systematic

## Abstract

**Background:**

The study aims to evaluate the effectiveness and safety of acupuncture therapy for elderly patients with coronavirus disease 2019 (COVID-19).

**Methods:**

Relevant articles from December 2019 to December 2020 will be searched in the following electronic databases: Medline, PubMed, EMBASE, Web of Science, Cochrane Library, China National Knowledge Infrastructure (CNKI), China Biomedical Literature Database (CBM), and China Scientific Journals Database. All published randomized controlled trials (RCTs) and credible clinical observations about this topic will be included. Two independent researchers will operate article retrieval, duplication removing, screening and data analysis by EndNote X9.0 and Stata 15.0. We will use the Cochrane risk of bias tool for randomized trials to assess the risk of bias of included studies. Meta-analysis, subgroup analysis, and/or descriptive analysis will be performed according to the data conditions included.

**Results:**

High-quality synthesis and/or descriptive analysis of current evidence will be provided from mortality rate, cure rate, C-reactive protein (CRP), creatine, troponin, aspartate aminotransferase, alanine aminotransferase, and improvements in chest CT scans, clinical symptoms (including fever, fatigue, cough, nausea, vomiting and diarrhea) and the side effects of acupuncture.

**Conclusion:**

This study will provide evidence of whether acupuncture is an effective and safe intervention for the elderly with COVID-19.

**PROSPERO registration number:**

CRD42020225245.

## 1 Introduction

In December 2019, the first case of COVID-19 was found in Wuhan, China.^[1,2]^ Subsequently, COVID-19 quickly broke out around the world, causing serious complications and death, and causing panic and anxiety among people all over the world.^[3,4]^ By December 2020, China reported the cumulative number of infections was nearly 70 million, and the cumulative death toll was nearly 1.6 million.

Current studies have found that COVID-19 poses a serious health threat to the elderly.^[5–10]^ Elderly men with higher body mass index, high respiratory rate and underlying diseases (such as hypertension, diabetes, cardiovascular disease and chronic obstructive pulmonary disease) are more likely to develop severe COVID-19 infection. The main symptoms are fever and dyspnea. Laboratory tests showed that the number of white blood cells was higher in the elderly group than in the young patients, and the number of lymphocytes was lower in the young patients.^[10,11]^ The lack of effective treatments for COVID-19 at home and abroad, especially for the elderly, which has made the COVID-19 pandemic the most serious global public health crisis.^[12–14]^

As is known to all, COVID-19 has been well controlled in China, mainly due to the joint efforts of Chinese and western medicine, especially Chinese medicine, which has made outstanding contributions to improving patients’ symptoms and promoting recovery.^[15–18]^ Acupuncture, as one of the traditional Chinese medicine treatment, has the advantage of quick effect, simple operation, and less pain, etc. Studies have shown that it can improve symptoms of dyspnea, relieve anxiety, treat insomnia, vomiting, and diarrhea, etc. so it has been widely used in the prevention, treatment and rehabilitation of patients with COVID-19.^[19–24]^

According to published studies, there is a lack of high-quality evidence on acupuncture for the elderly with COVID-19. Therefore, this systematic review aims to evaluate the effectiveness and safety of acupuncture in the treatment of elderly patients with COVID-19.

## 2 Methods

### 2.1 Study registration

This systematic review protocol has been registered in the PROSPERO network (No. CRD42020225245). And it will follow recommendations outlined in The Cochrane Handbook of Systematic Review of Interventions.

### 2.2 Types of studies

RCTs and credible clinical observations without any limitation of blinding or publication language will be included. Animal studies, reviews, case reports, meta-analyses, and republished papers will be excluded.

### 2.3 Types of participants

Elderly patients (≥60 years) diagnosed with COVID-19 and receiving acupuncture treatment will be included, with no restrictions on gender, race, nationality or disease.^[25]^

### 2.4 Types of interventions and comparisons

Observation group: Types of acupuncture, moxibustion, electroacupuncture, fire needle, warming needle moxibustion, acupoint injection and auricular therapy will be included.

Control group: Any method other than acupuncture will be included, such as placebo, Traditional Chinese medicine, Western medicine and non-intervention, etc.

### 2.5 Types of outcomes.

Primary outcomes will include mortality rate, cure rate, C-reactive protein (CRP), creatine, troponin, aspartate aminotransferase, alanine aminotransferase, and improvements in chest CT scans. Secondary outcomes will include the disappearance time of clinical symptoms (including fever, fatigue, cough, nausea, vomiting and diarrhea) and the side effects of acupuncture.

### 2.6 Search methods for identification of studies

Relevant articles from December 2019 to December 2020 will be searched in the following electronic databases: Medline, PubMed, EMBASE, Web of Science, Cochrane Library, CNKI, CBM, China Scientific Journals Database, etc. The complete Medline search strategy is summarized in Table 1. According to different databases, keywords can be combined with free words for comprehensive search.

**Table 1:** Medline search strategy.

Number	Search items
#1	MeSH major topic: Coronavirus disease 2019
#2	MeSH major topic: COVID-19
#3	MeSH major topic: 2019-nCoV
#4	MeSH major topic: 2019 novel coronavirus
#5	MeSH major topic: novel coronavirus pneumonia
#6	MeSH major topic: novel coronavirus
#7	MeSH major topic: acupuncture
#8	MeSH major topic: moxibustion
#9	MeSH major topic: electroacupuncture
#10	MeSH major topic: fire needle
#11	MeSH major topic: acupoint injection
#12	MeSH major topic: auricular point
#13	MeSH major topic: warming needle moxibustion
#14	MeSH major topic: adult
#15	MeSH major topic: aged
#16	MeSH major topic: aging
#17	MeSH major topic: older adult
#18	MeSH major topic: elderly
#19	MeSH major topic: agedness
#20	MeSH major topic: gerontism
#21	#1 or #2 or #3 or #4 or#5 or #6
#22	#7 or #8or #9 or #10 or #11 or #12 or #13
#23	#14 or #15 or #16 or #17 or #18 or #19 or #20
#24	#21 and #22 and #23

### 2.7 Data collection and analysis

#### 2.7.1 Selection of studies

**Figure 1:**
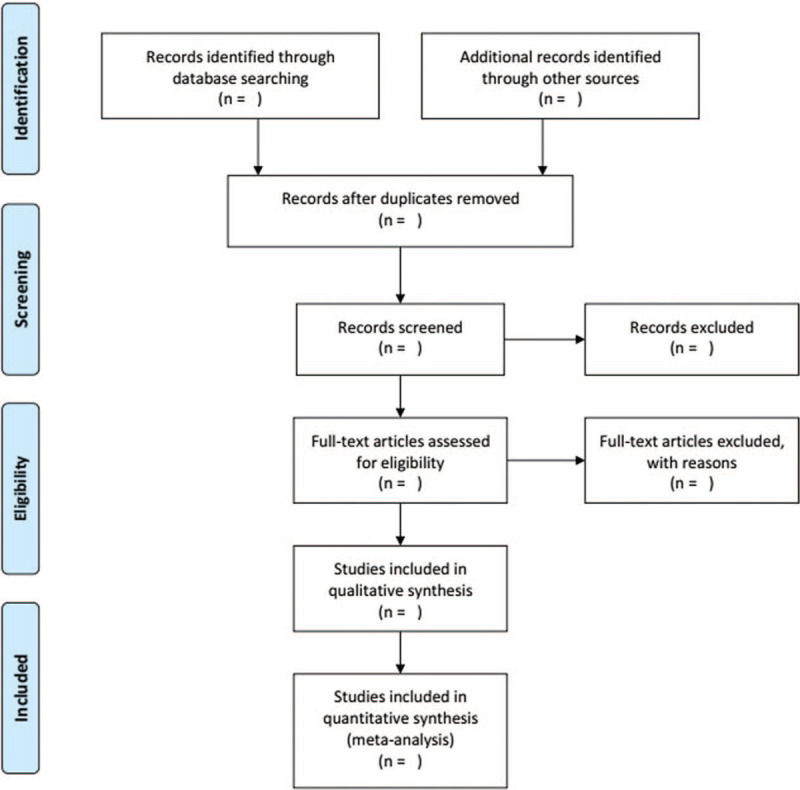
The PRISMA flow diagram.

Two reviewers (QCX and HWG) will screen the study independently and check the results with each other. When disagreements occur, the third examiner (YZ) will make the final decision. If necessary, they will read the full text of all included study. If complete literature or relevant data is not available, we will contact the corresponding author. The filter operation process will be shown in the Preferred Reporting Items for Systematic Reviews and Meta-Analyses Protocols flow diagram (Fig. 1).

#### 2.7.2 Assessment and quality of included studies

Two reviewers (XY and YZ) will assess the quality and risk of bias of the included articles according to the Cochrane Handbook. Quality assessment of included studies contains random sequence generation, allocation concealment, blindness to outcome assessment, blindness of participants and personnel, incomplete outcome data, selective reporting and other biases. Based on the research assessment of these seven areas, they will be classified as “low risk”, “high risk” or “unclear risk” bias. A third reviewer (QCX) will be consulted if there is an assessment disagreement.

#### 2.7.3 Data extraction

Two independent reviewers (QCX and HWG) will extract the evaluated article data with a standardized data extraction form, and when they have differences of opinion, they will discuss them with a third reviewer (YXM) if they can not resolve them. The data will be recorded onto an electronic form, including general information about the article (first author, title, year and language), inclusion and exclusion criteria, baseline of the study (sample size, sex ratio, age and disease stage), interventions used in the observation and control groups, and outcome measures.

#### 2.7.4 Measures of treatment effect

All efficacy data will be transferred to Stata 15 for analysis and synthesis. For continuous results, the data will be presented by mean difference (MD) or standard mean difference (SMD) with 95% confidence intervals (CIs). When dichotomous data exists, risk ratio (RR) with a 95% CI will be used. When binary data exists, the RR form will be changed for analysis.

#### 2.7.5 Dealing with missing data

If the data is lost in the literature, we will contact the corresponding author by email or other means. If the missing data is not available, we will exclude these articles and integrate the rest of the research.

#### 2.7.6 Assessment of heterogeneity

The included studies will use the chi-square test and Q test to evaluate the heterogeneity. If I^2^ < 50%, the heterogeneity of the text will be ignored and the fixed-effect model will be used. If I^2^ ≥ 50%, statistical heterogeneity will be considered to be significant, the random-effect model will be adopted.^[26]^

#### 2.7.7 Assessment of reporting bias

We will use the funnel plot and Egger test to assess publication bias if >10 articles are included.

#### 2.7.8 Data synthesis

We will use the Stata 15.0 software for meta-analysis. If the clinical and methodological heterogeneity is low, fixed-effect models will be used. If heterogeneity is significant, we will use the random-effects model and perform subgroup analysis or descriptive analysis.

#### 2.7.9 Subgroup analysis

Subgroup analysis will be performed based on the results of the data synthesis, if necessary, we will conduct subgroup analysis of any source of heterogeneity.

#### 2.7.10 Sensitivity analysis

The sensitivity analysis will be performed by using Leave 1 out. We will plan to conduct a sensitivity analysis of the main results to figure out whether the results are affected by the use of different analytical methods (random-effects model or fixed-effects model) and to confirm the robustness of our results.

#### 2.7.11 Quality of evidence

Two reviewers (JX and YPL) will independently evaluate the quality of evidence by using the Grading of Recommendations Assessment, Development and Evaluation (GRADE). We will evaluate the quality of evidence as “high,” “moderate,” “low,” or “very low” according to five parameters (publication bias, indirectness, inconsistency, imprecision, and study limitations). A third reviewer (QCX) will be consulted if quality ratings are not consistent between the two reviewers.

#### 2.7.12 Ethics and dissemination

Because no data in this review are relevant to individual patients, ethical approval is not required for this study.

## 3 Discussion

The highly infectious and pathogenic characteristics of COVID-19 have made it spread rapidly around the world. It not only endangers the health of the public, but also causes major damage to the world's economic development. Elderly people with poor resistance, in particular, are the preferred targets for COVID-19.

Therefore, how to protect the lives of the elderly in the epidemic situation has become a research hotspot in the current medical field. As one of the traditional Chinese medicine treatment methods, acupuncture can not only be used to prevent the disease before it occurs, but also be used as an auxiliary treatment after it occurs. If there is evidence that acupuncture works for the elderly with COVID-19, it will benefit the world. Thus, a systematic review about it is urgently needed. This study could provide evidence for acupuncture treatment on elderly patients with COVID-19 and help clinicians make decisions.

## Author contributions

**Conceptualization:** Qingchang Xia, Huawei Gao, Yue Zhou.

**Data curation:** Huawei Gao, Xiao Yan, Yue Zhou.

**Investigation:** Qingchang Xia, Yue Zhou.

**Methodology:** Jin Xian, Xiao Yan.

**Project administration:** Qingchang Xia.

**Supervision:** Qingchang Xia, Huawei Gao.

**Validation:** Jin Xian, Yuxai Ma.

**Visualization:** Qingchang Xia, Yunping Lu.

**Writing – original draft:** Huawei Gao.

**Writing – review & editing:** Jin Xian, Yuxai Ma.
